# The contemporary management of parastomal varices by interventional radiology: a systematic review

**DOI:** 10.1186/s42155-025-00615-1

**Published:** 2025-11-13

**Authors:** Jack M. Bell, Hugo C. Temperley, Benjamin M. Mac Curtain, Nicholas A. Clausen, Robert S. Doyle, Noel E. Donlon, Kevin Sheahan, Michael J. Lee

**Affiliations:** 1 Department of Radiology, Beaumont Hospital, Dublin, D09V2N0 Ireland; 2 Department of Radiology, St. James’ Hospital, Dublin, D08NHY1 Ireland; 3 Department of Urology, Northwell Health, New Hyde Park, New York, USA; 4 Department of Surgery, Waterford University Hospital, Waterford, X91 ER8E Ireland; 5 Cardiology Department, Mater Misericordiae University Hospital, Eccles St., Dublin, D07 R2WY Ireland; 6 Department of Surgery, St. James’ Hospital, Dublin, D08NHY1 Ireland; 7 Royal College of Surgeons in Ireland, Dublin, D02 YN77 Ireland

**Keywords:** Parastomal varices, Portal hypertension, Interventional radiology, Transjugular intrahepatic portosystemic shunt (TIPS)

## Abstract

**Background:**

Parastomal varices are a rare but serious complication in patients with portal hypertension, characterised by bleeding that can be life-threatening in a predominantly comorbid population. Traditional surgical approaches to managing parastomal varices are associated with high morbidity and recurrence rates, prompting increased interest in minimally invasive techniques. This systematic review aims to evaluate the efficacy and safety of interventional radiology (IR) procedures, including transjugular intrahepatic portosystemic shunt (TIPS), sclerotherapy and embolisation, in managing parastomal varices.

**Methods:**

A comprehensive literature search was conducted across multiple databases, including MEDLINE, EMBASE and Web of Science, to identify studies published up to January 2025 that reported IR interventions for parastomal varices. Data were extracted on patient demographics, procedural success, recurrence rates and complications. A pooled proportions meta-analysis was performed.

**Results:**

Five studies, encompassing 45 patients, met the inclusion criteria. The pooled technical success rate of IR procedures was 91.3%, with a clinical success rate of 80.5% over a mean follow-up of 618.4 days. The pooled mean proportion of rebleeding, predominantly minor and non-life-threatening, was 36.4%. TIPS showed the highest efficacy, but is traditionally associated with increased procedural risks compared to other interventional radiology methods.

**Conclusion:**

IR offers a highly effective and safe alternative to traditional surgical management for parastomal varices in contemporary terms. The low recurrence and complication rates highlight the potential of IR ab initio as a first-line treatment; consequently, we advocate for its use, particularly in patients unsuitable for surgery in the minimally invasive era.

**Systematic review registration:**

PROSPERO CRD42024627470.

## Introduction

Parastomal varices are extraperitoneal, ectopic, abnormally dilated veins which develop at stoma sites. These varices can be classified as type 1, also known as non-occlusive or oncotic type, due to portal hypertension, or type 2, also known as occlusive type, which is caused by venous occlusion [1]. Parastomal varices develop in an estimated 50% of patients with concomitant portal hypertension, with over a quarter of these patients presenting with a parastomal variceal bleed [1]. Parastomal varices are classically associated with patients who have developed primary sclerosing cholangitis (PSC) with concomitant ulcerative colitis and who have undergone ileostomy formation following proctocolectomy [2].

Parastomal variceal bleeding (PVB) is the most common presentation to emergent care postoperatively in this cohort, with a reported incidence of 27% [3]. First described in the 1960 s [4], these bleeds typically occur 48 months post-stoma formation in these patients and can result in significant blood loss, carrying a 3–4% mortality rate per bleed [5]. Many bleeds can be conservatively managed with manual compression or local epinephrine. Still, if these fail, other options for PVB include pharmacological management, other local therapies, percutaneous transvenous embolisation, transjugular intrahepatic portosystemic shunt (TIPS) and stoma re-siting. Patients bleeding from parastomal varices are often poor surgical candidates in both emergent and elective settings, with comorbidities such as hypoalbuminaemia and anaemia conferring increased risks of poor healing rates and postoperative mortality. Concomitant cirrhosis has been shown to further worsen morbidity in up to 83% of these patients, increasing the length of stay in postoperative patients due to ascites, renal failure, bleeding and infection [6].

Thus, image-guided interventions have been increasingly employed in managing parastomal variceal haemorrhage. TIPS, percutaneous antegrade transhepatic venous embolisation with various agents, and local transvenous embolisation of parastomal varices remain the favoured minimally invasive approaches in patients with refractory or recurrent bleeding [7].

TIPS is an efficient measure in addressing portal hypertension and preventing rebleeding, with success rates as high as 95% when combined with percutaneous antegrade transhepatic venous obliteration (PATVO). However, minimally invasive interventions are more favourable in this cohort due to the risk of hepatic encephalopathy and the need for general anaesthesia [8]. While TIPS is highly successful, the literature still quotes a 20% rebleed rate [9].

Due to the numerous minimally invasive techniques employed and the limited number of case reports and case series, evidence is scarce regarding the optimal management of PVB. This systematic review, the first of its kind, aims to assess minimally invasive options in terms of technical success, clinical success and patient outcomes.

## Methods

### Study design and reporting guidelines

This study is a systematic review of non-randomised trials, following the Preferred Reporting Items for Systematic Reviews and Meta-Analyses (PRISMA) reporting guidelines [10].

### Search strategy

The following databases were searched as part of the systematic review in January 2025: MEDLINE, EMBASE and Web of Science. The search combined terms related to the condition (‘parastomal varices,’ ‘stomal varices,’ ‘peristomal varices’ and ‘ectopic varices’) with interventional procedures (‘interventional radiology,’ ‘transjugular intrahepatic portosystemic shunt,’ ‘TIPS,’ ‘sclerotherapy’ and ‘embolisation’). Additional outcome-related terms such as ‘technical success,’ ‘clinical success,’ ‘rebleeding,’ ‘recurrence’ and ‘complications’ were included to refine the search. Boolean operators (AND/OR) were used to combine terms appropriately. The last date of the search was 5 January 2025. Grey literature was also searched to identify other suitable publications.

### Inclusion criteria

Studies in English were assessed for eligibility based on the following inclusion criteria: studies outlining cases of bleeding parastomal varices and the techniques used to combat this were included in our analysis. Conference abstracts, case reports and case series (comprising fewer than five patients) were excluded.

i. Study design:
  - Cohort studies and case-control studies
  - Original research with a sample size greater than or equal to 5 patients
  - Patients diagnosed with parastomal varices associated with portal hypertension.
ii. Interventions:
  - Interventional radiology procedures, including but not limited to TIPS, sclerotherapy and embolisation.
iii. Outcomes:
  - Technical success: defined as the confirmed obliteration of the causative vessel to the parastomal varices, confirmed on imaging at the time of the procedure or in the immediate follow-up.
  - Clinical success: defined as no significant re-bleeding in the 2 weeks following their procedure.
  - Re-bleeding: defined as post-procedure stomal bleeding requiring hospitalisation or transfusion.
  - Follow-up: mean follow-up period (days)

### Study selection, data extraction and critical appraisal

A database was compiled using EndNote X9™. Two reviewers (JB and HT) independently screened studies, removing duplicates, then assessing titles, abstracts and full texts based on predefined criteria. Excluded studies were categorised by exclusion reason.

The Cochrane Collaboration’s screening and data extraction tool, Covidence, was used [11] to extract and store data efficiently. Two reviewers (JB and HT) independently collected data using the following headings: study details, study design, population, intervention, comparison groups and outcomes. Discrepancies in study selection or data extraction were resolved through discussion with a senior reviewer (KPS).

A critical appraisal of the methodological quality and risk of bias in the included studies was performed. The critical appraisal was completed independently by two reviewers. Quality assessment of the included studies was performed using a modified Newcastle–Ottawa scale (NOS) risk of bias tool [12], and the results were tabulated. We calculated the risk of bias (ROB) for the specific organ system and the total throughout all the studies. This assessment tool categorises each study as ‘satisfactory’ or ‘unsatisfactory’ across various categories. We assigned stars to evaluate study quality: 7 stars indicate ‘very good’, 5–6 stars indicate ‘good’, 3–4 stars indicate ‘satisfactory’ and 0–2 stars indicate ‘unsatisfactory’. Two reviewers (JB and HT) independently completed the critical appraisal. Once again, a third reviewer (KPS) was asked to arbitrate in cases of discrepancies in opinion.

### Statistical analysis

Statistical analysis was conducted using Stata 17. Proportions were pooled using the ‘metaprop’ function and hazard ratios (HR) with 95% confidence intervals (CI) were analysed via generic inverse variance methods. Studies reporting HR with CI or *p*-values were included. Means and standard deviations (SD) were recorded or estimated from medians and ranges using Hozo et al. [13]. Heterogeneity was assessed using *I*^2^, with > 50% indicating substantial heterogeneity. Statistical significance was set at *p* < 0.05, and a random-effects model was used throughout.

### Systematic review registration

Our systematic review was registered on PROSPERO in December 2024 (ID: CRD42024627470).

## Results

**Fig. 1:**
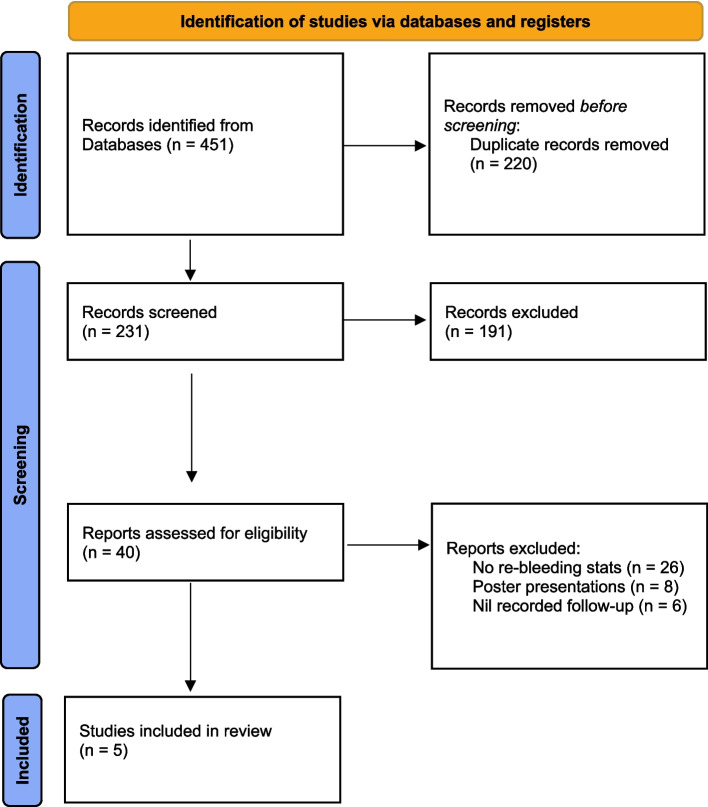
Prisma statement

The literature search described yielded 451 results (see Fig. 1). Following the removal of 220 duplicates, 231 studies were screened. Following the initial screen, 40 full texts were reviewed and assessed for eligibility. Five studies met the eligibility criteria and were included in our analysis. All five provided sufficient statistical data to be included in our quantitative analysis.

The five included studies [14–18], published from 2008 to 2022, were published in Canada [14], the USA [1, 15, 17], France [16] and Germany [18]. The total sample size across all studies was 45 patients, with a combined 51 procedures performed; this included recorded re-intervention procedures where re-bleeding occurred. The mean age of the patients was 61.96 years, with 27 males and 18 females included. All studies were retrospective, comprising case series and reviews, covering periods ranging from a few years to a decade. The mean patient follow-up time was 618.4 days (range 392.4–793.9, SD 174.4).

All patients had varying degrees of liver failure, summarised in 4/5 studies using the Model for End-stage Liver Disease (MELD) score (average MELD score was 13.4). Additionally, the cause of cirrhosis was documented across all patients included in this review: non-alcoholic fatty liver disease (NAFLD) and alcohol accounted for 31.1%; hepatitis C virus (HCV) represented 6.7%; primary sclerosing cholangitis (PSC), primary biliary cirrhosis (PBC) and cryptogenic liver disease each represented 4.4%; portal venous thrombosis, liver metastases, total parenteral nutrition (TPN) and drug-induced represented 2.2%, respectively. Two patients had cirrhosis of unknown origin, and a further two were non-cirrhotic (4.4% respectively) (Tables 1, 2 and 3).

**Table 1:** Study characteristics

Author	Year	Location	Sample size	Procedures performed	Age	Gender distribution (M:F)	Design	Period
Nadeem	2022	Canada	12	17	Mean 60.1 (SD 17.5)	(9:3)	Retrospective case series	2016–2021
Pabon Ramos	2013	USA	7	8	Mean 51.1 (SD 21.6)	(3:4)	Retrospective	2000–2008
Thouveny	2008	France	7	7	Mean 69 (range 50–78)	(5:2)	Retrospective case series	1998–2006
Young	2018	USA	10	3 sclerotherapy, 5 TIPs, 2 both (10 total procedures)	Mean 62.6 (range 44–84)	(4:6)	Retrospective review	2006–2016
Kaczmarek	2021	Germany	9	9	Mean 67 (range 37–79)	(6:3)	Retrospective	2014–2020

*TIPS *Transjugular intrahepatic portosystemic shunt

**Table 2:** Cirrhosis status of patients included

Author	Cirrhosis status
Nadeem et al.	MELD mean 12.3 (± 4.2)
Pabon Ramos et al.	MELD mean 12 (± 6.5)
Thouveny et al.	Not specified
Young et al.	MELD mean 18.2 (sclerotherapy), 13.3 (TIPS) Child–Pugh 9.2 (sclerotherapy), 7.7 (TIPS)
Kaczmarek et al.	MELD mean 11, Child–Pugh 6

* MELD *Model for End-stage Liver Disease

**Table 3:** Causes of cirrhosis

Cirrhosis cause	n (total = 45)
NAFLD	14
Alcohol	14
HCV	3
PSC	2
PBC	2
TPN	1
Cryptogenic	2
Portal vein thrombosis	1
Liver Mets	1
Drug Induced	1
Unknown	2
Non cirrhotic	2

*NAFLD *Non-alcoholic fatty liver disease

*HCV *Hepatitis C Virus

*PSC *Primary Sclerosing Cholangitis

*PBC *Primary Biliary Cirrhosis

*TPN *Total parenteral nutrition

### Technical aspects of procedures

The technical aspects of the procedures are shown in Tables 4 and 5. Studies employed different percutaneous access techniques, where stated, with the target always being branches of the superior mesenteric vein (SMV); Young [17] and Kaczmarek et al. [18] did not specify their target for direct techniques. The access for the procedures also varied depending on the technique used, including external jugular access for TIPS, percutaneous portal venous access and direct local percutaneous access of the varices.

**Table 4:** Technical details of procedures

Author	Access	Target
Nadeem	Percutaneous portal venous	Branch of SMV
Pabon Ramos	Percutaneous systemic outflow of varix	Branch of SMV
Thouveny	Percutaneous systemic outflow of varix	Branch of SMV
Young	External jugular and percutaneous access of varix	Not stated
Kaczmarek	External Jugular	TIPS, direct techniques not stated

*SMV *Superior mesenteric vein

**Table 5:** Details of embolic agents used across studies

Embolic agent	*N*	% usage of procedures
Coil	17	33.3
Thrombin	5	9.8
NBCA/glue polymerisation	20	39.2
STS Foam	7	13.7
Embozene particles	4	7.8
Ethanol in lipiodol	3	5.9
Sotradol in lipiodol	2	3.9
Gelfoam sclerosant	2	3.9
Sodium morrhuate	1	2.0

*NBCA *N-Butyl Cyano-acrylate

*STS *Sodium Tetradecyl Sulphate

A wide range of embolic agents was used across the studies. A total of 51 procedures were performed (including both initial procedures and repeat procedures), and operators used a variety of combinations of embolic agents. The use of each agent is described in Table 5, where the usage rate for each agent is shown. Note that these agents were rarely used in isolation. The most commonly used agent was NBCA/glue polymerisation, which was used in 39.2% of cases. This was followed by coil embolisations, used in 33.3% of cases. STS foam was the third most commonly used, being employed in 13.7% of cases. Thrombin (9.8%), embozene particles (7.8%), ethanol in lipiodol (5.9%), sotradol in lipiodol (3.9%), gelfoam sclerosant (3.9%) and sodium morrhuate (2.0%) accounted for the remaining embolic agents.

Several interventional techniques were employed, as shown in Table 6.

**Table 6:** Procedural techniques performed and re-bleeding rates of initial procedures (i.e. bleed within initial 24 h)

Technique	*N*	Early re-bleed	Initial re-intervention
PATVO	12	3	3 × PATVO
US-guided percutaneous cannulation of portal inflow system and sclerotherapy	17	2	1 × repeat, 1 × deceased
TIPS	10	2	TIPs revision + coil
TIPS with percutaenous sclerotherapy	2	0	N/A
TIPS with coil + cyanoacrylate	1	0	N/A
TIPS with coil embolisation	3	0	N/A

*PATVO *Percutaneous antegrade transhepatic venous obliteration

*TIPS *Transjugular intrahepatic portosystemic shunt

#### Percutaneous US guided

The most common technique employed was US-guided percutaneous cannulation of the portal inflow system, combined with sclerotherapy, accounting for 37.8% (17/45) of the initial procedures. This access was achieved by accessing a systemic venous branch of the varix through percutaneous access close to the stoma. The micropuncture needle was then directed retrogradely to access the bleeding varix. Occasionally, the access site had to be abandoned in favour of another due to persistent vasospasm. A combination of embolising agents was then injected at the point of portal flow reversal.

#### PATVO

The following most commonly used individual technique was PATVO, accounting for 26.7% (12/45) of initial procedures. Using ultrasound guidance, access to peripheral segments 5 or 6 of the portal vein branch was obtained percutaneously. Following the venograms, branches of the SMV were identified, and branch points of the associated varices were noted. After negotiating these and subselecting the bleeding varix, embolisation was performed using a combination of embolic agents.

#### TIPS and combined interventions

There were several combinations of TIPS procedures, with or without embolisation/sclerotherapy, with the TIPS group as a whole accounting for 35.6% (16/45) of initial procedures. Two of these were secondary procedures after failed initial local IR attempts. Of these latter two salvage procedures, both patients had cirrhosis secondary to NAFLD and had initial percutaneous sclerotherapy injections using STS foam. Following 2 and 3 repeat procedures, respectively, these patients went on to have subsequent TIPS procedures due to recurrent bleeding. A further three patients underwent subsequent TIPS procedures in the longer-term follow-up, all of whom had an initial PATVO.

### Re-bleeding

The rebleeding rates at any stage of follow-up (i.e. including early [< 24 h] and late rebleeds) were calculated for the corresponding procedures and are presented in Table 7. Among the techniques employed in multiple patients, PATVO represented the highest anytime re-bleeding rate of 47.1%, with TIPS procedures showing a re-bleeding rate of 20% across all procedures. Notably, TIPS combined with local techniques resulted in a rebleeding rate of 0%. US-guided percutaneous cannulation of the portal inflow system and sclerotherapy showed a rebleed rate of 33.3%. Repeat intervention can also be seen in Table 7, with most rebleeders having a repeat procedure of their initial treatment. Rescue therapy, where continuous bleeding was experienced despite re-intervention, was TIPS across all studies described.

**Table 7:** Any time rebleeding and subsequent reintervention. Re-bleeding rates calculated based on all procedures

Technique	All re-bleed	Re-bleed rate	Re-intervention
PATVO	8	47.10%	5 × PATVO (1 requiring subsequent TIPS), 2 × TIPS
US guided percutaneous cannulation of portal inflow system and sclerotherapy	5	24%	3 × repeat US guided percutaneous, 1 × transhepatic embolisation, 1 × conservative management
TIPS	0	0%	N/A
TIPS with embolization of femoral varices	0	0%	N/A
TIPS with percutaneous sclerotherapy	0	0%	N/A
Gelfoam + sclerosant embolization	1	100%	2 × repeat percutaneous sclerotherapy (both subsequently requiring TIPS)
Coil embolisation + sclerotherapy	1	0	N/A

### Procedural outcomes

Table 8 outlines the outcomes across all studies, showing a high technical success rate, as defined above, across all techniques utilised in each study. We calculated the pooled mean proportion for each of the following outcomes, allowing us to assess weighted averages for the patients.

i) Technical success
  The pooled mean proportion for technical success was 91.3% (95% CI 0.826–0.972). Two of the five studies achieved a 100% technical success rate. Technical failure was attributed to inaccessible vasculature in 2 patients. In Pabon Ramos et al., the failure was due to the inability to negotiate a hairpin bend between a peristomal varix and the portal inflow vein. In Thouveny et al., the varix collapsed under simple pressure from the US probe. Despite multiple attempts, the needle could not be appropriately positioned within the varix.
ii) Clinical success
  On analysis of clinical success, the pooled mean proportion was 80.5% (95% CI 0.692–0.897). This varied across studies, with Kaczmarek et al. [18] achieving the highest clinical success rate of 100% and Young et al. [17] achieving the lowest clinical success rate of 70%.
iii) Late re-bleeding
  The re-bleeding rate was defined as bleeding experienced at any point in clinical follow-up, i.e. beyond that of clinical success. The pooled mean proportion was calculated to be 36.4% (95% CI 0.244–0.493), with an average time to re-bleed of 365.78 days (range 128–760.4, SD 234.6).

**Table 8:** Clinical outcomes

Author	Technical success	Clinical success	Re-bleeding	Time to re-bleed (mean days)	Mortality (procedure)	Mean follow-up (days)
Nadeem	100%	82%	8/17 (47.1%)	131.4 (SD 136.3) where reported	0%	392.4
Pabon Ramos	88%	88% (3 × rebleeds, 26 days, 45 days, 313 days)	3/8 (37.5%)	128 (SD 131.0)	0%	685.9
Thouveny	85.70%	71.40%	2/7 (28.6%)	352.8 (11.6 months)	0%	428.9
Young	100%	7/10 (70%—note 2/10 went on to have TIPs due to persistent bleed)	3/10 (30%)	456.3 (15 months)	0%	793.9
Kaczmarek	100%	100% (9/9)	2/9 (22.2%)	760.4 (25 months)	0%	790.9

### Morbidity and mortality

Mortality across all procedures was low, with all five studies having procedural mortality rates of 0%.

Morbidity of procedures was reflected in the early re-bleeding rates, as seen in Table 6. PATVO patients experienced three early rebleeds (a 25% early rebleed rate), and each rebleed was treated with a subsequent PATVO. US-guided percutaneous cannulation of the portal inflow system at the stoma combined with sclerotherapy had two early re-bleeds (11.8% early re-bleed rate); one had a repeat procedure, and one died. Patients who underwent TIPS procedures alone had two early rebleeds, which were attributed to TIPS dysfunction; these were subsequently treated with TIPS revision and coil embolisation. It should be noted that the re-bleeds in the TIPS alone group appeared at 2 and 48 months, therefore lying beyond our ‘clinical success’ mark.

### Quality of studies

Regarding quality assessment, one study was rated ‘very good’, three were rated ‘good’, one was rated ‘satisfactory’ and no studies were rated ‘unsatisfactory’ when scored using the NOS risk of bias assessment. Supplementary material 1 summarises the results of the risk of bias assessment.

## Discussion

Our systematic review aimed to assess the efficacy and safety of interventional radiology techniques, including sclerotherapy and embolisation, as alternatives to traditional surgical management of PVB. The findings suggest that these minimally invasive procedures offer promising clinical success, with 72% of patients experiencing no further significant bleeding 2 weeks post-procedure. Furthermore, 33.08% of patients experienced late rebleeding, which on average occurred 365.7 days post-procedure. We can see the potential benefit when compared to traditional surgical portosystemic shunting or embolisation alone, which leaves patients with approximately a 50% chance of rebleeding [9].

Evidence regarding the success of minimally invasive techniques, such as embolisation and sclerotherapy, is mainly found in case reports and case series [3, 4, 19–28]. The pooled technical success mean proportion found in our results was notably high, 93.5%. This success rate is consistent with the literature, as evidenced by the 94% technical success rate reported by Macedo et al. in their case series [21]. Where technical success was not achieved, it was attributed to inaccessible vasculature, with the superior mesenteric vein being the primary target in 96% of cases, as is commonly observed [3, 22]. However, while these results are encouraging, the evidence base remains limited and heterogeneous, and our findings should highlight the need for further research into minimally invasive techniques as a first-line therapy. No statistically significant heterogeneity was detected (*Q* < df; *I*^2^ ≈ 0%), although given the small number and size of included studies, this likely reflects limited power rather than true homogeneity.

There was a low associated mortality rate (0%), which was challenging to estimate due to the scarcity of extensive cohort studies available. Of the available case reports and case series, deaths occurring peri-procedurally are typically secondary to decompensation of respiratory/hepatic/cardiac function rather than being procedure-specific [23–27].

While techniques such as embolisation demonstrate promising clinical and technical success rates, our results indicate that 34.8% of patients will experience recurrent bleeding at an interval of 7–8 months (211 days) post-procedure. Notably, the patients included in this review were followed up for an average of 618.4 days, which may have limited our ability to determine the late rebleed risk confidently. However, it may be negatively biased due to the short follow-up period. In 2012, Pennick et al. found that 81% of patients undergoing local surgical management of varices bled again, compared to 45% in patients who underwent percutaneous embolisation [9]. Notably, combinations of sclerotherapy or embolisation with TIPS yielded the best success rates in terms of re-bleeding, highlighting the potential role that interventional radiology techniques may play in the future treatment of this condition. However, performing TIPS remains a clinical decision and is patient-specific due to the associated risk of hepatic encephalopathy [28]. Furthermore, the lack of standardisation of follow-up poses difficulties in accurately assessing long-term outcomes.

The highest success rates, i.e. with the lowest re-bleeding rates, were observed in the TIPS procedures. The difficulties associated with TIPS are well documented, and many patients are ineligible due to contraindications. Across all included procedures, TIPS was most often used as a last resort or rescue therapy, where it was employed when persistent bleeding occurred following less invasive techniques. With these procedures, there was often variation in the significance of the patient’s liver disease. Therefore, some studies experienced particularly troublesome bleeders, which may skew re-bleeding rates.

Several limitations were identified within this review. Firstly, due to the rarity of the disorder, there were minimal numbers available on reviewing the literature. The use of less invasive techniques is a relatively new tactic, and as a result, the number of reported cases is currently lacking. Additionally, the heterogeneity of procedures employed leads to inconsistencies in practice. There is currently no gold standard of treatment, and as such, a mix of techniques is often used. Finally, there is considerable heterogeneity of data, with various definitions of success, complications and follow-up.

Future research should focus on prospective head-to-head comparisons of interventional radiology techniques to define optimal treatment strategies. Given the rarity and acute presentation of parastomal variceal bleeding, this may be challenging. Long-term outcomes and quality-of-life comparisons with conventional surgery are also needed to clarify the durability and overall benefit of these interventions.

In conclusion, contemporary interventional radiology techniques represent a significant advancement in managing parastomal varices. These methods may effectively control variceal bleeding with acceptable recurrence and complication rates, positioning them as viable alternatives to traditional surgical approaches and their associated morbidity and mortality. Whilst there is a definite paucity in reporting within this area, the continued evolution and refinement of these techniques should promise to improve patient outcomes and reduce disease burden. We believe this emphasises the need for larger, prospective, multicentre studies to offer more definitive evidence for the use of IR as first-line treatment in this cohort.

## Data Availability

The datasets used and/or analysed during the current study are available from the corresponding author on reasonable request.
